# The court’s judgment on the true identity of the author responsible for identity fraud and the consequent batch retraction of Iranian’s papers in the journal of diagnostic pathology

**DOI:** 10.1186/s13000-024-01459-4

**Published:** 2024-02-12

**Authors:** Aram Mokarizadeh

**Affiliations:** Independent Researcher, Tehran, Iran

**Keywords:** Retraction, Misconduct, Submitting author, Court, Identity fraud

## Abstract

This letter concerns retracted papers published in the Journal of Diagnostic Pathology, where my name was misused as the author or corresponding author without my permission or knowledge. Considering that all misconducts were directed by an author during initial manuscripts’ submissions, I opened a case in Iran’s Cyber Police (FATA) to unravel the true identity of the submitting author. After Cyber Police’s report revealed the true identity of the submitting author, the court started a thorough investigation and finally convicted the submitting author for identity fraud and data forgery through creating and using fake email addresses.

This clarification letter concerns the batch retraction of Iranian’s papers published in the Journal of Diagnostic Pathology [1–5] in November 2016, particularly in which my name was misused as the author or corresponding author. However, in the published reports, authorship and peer-review manipulations had been stated as the main reasons underlying retraction.

Considering that all misconducts were directed by an anonymous author during initial manuscripts’ submissions without my permission or knowledge, I opened a case in Iran’s Cyber Police (FATA) to unravel the true identity of the submitting author.

Accordingly, after Cyber Police’s investigation revealed the true identity of the submitting author, I filed a complaint against the accused person (Mr. J. Javanbakht) for identity fraud in the General and Revolutionary Prosecutor’s Office (District 31 of Tehran, Iran). However, after several years of careful investigations accompanied by the opinions of the members of expert panels and Cyber Police’s report, the trial court (the Branch no.1040 of Criminal Court 2, Shahid Qodousi Judicial Complex in Tehran) convicted Mr. J. Javanbakht for the forgery of data by creating and using fake e-mail addresses attributed to reputable authors and reviewers (identity fraud) upon initial submission of manuscripts [1–13]. The trial court verdict was issued under no. 140,068,390,008,027,479 on 05/09/2021. Subsequently, the court of appeal (the Branch no.67 of Tehran Province Court of Appeal) decisively confirmed the trial court decision as the final verdict issued under no. 140,168,390,006,910,053, on 16/08/2022.

In this regard, along with a brief disclosure report published by Retraction Watch [14], a relevant statement has been published in the Journal of Tumor Biology where the disclosed information was duly confirmed following an investigation conducted by the Editor-in-Chief in collaboration with Iran embassy in Warsaw [15].

## Data Availability

Court verdicts that are officially translated and certified by the Iran Ministry of Foreign Affairs, can be shared by the Editor-in-Chief or the editorial office of *Diagnostic Pathology* upon reasonable request.
